# New Appliances and Things Medical

**Published:** 1900-12-15

**Authors:** 


### New Appliances and Things Medical.

[We shall be glad to receive, at our Office, 28 & 29 Southampton Street, Strand, London, W.C., from the manufacturers, specimens of all new preparations and appliances which may be brought out from time to time.]

### KOLE SPAR.
(Unique Manufacturing Company, Rotherham.)

Kole spar claims to be a "coal saver" of great economic merit. One sixpenny packet is said to save one quarter of a ton of coal. The method is to dissolve kole spar in water and sprinkle it over the coals before use. Less draught and less coal will be required to liberate the same amount of heat. It is difficult without elaborate experiments to prove that the above claims are accurate, but trying the effect in a rough-and-ready way, we certainly think that there was a decided economy in the burning of coal in any ordinary domestic open fireplace. It may be worth the householder's while to make the same experiments for himself, and if practically he finds there is a real economy to adopt so ingenious a method.

